# L´accouchement des grossesses gémellaires et pronostic materno-fœtal dans un Centre Universitaire Tunisien de niveau 3: étude rétrospective à propos de 399 cas

**DOI:** 10.11604/pamj.2020.36.237.19179

**Published:** 2020-08-03

**Authors:** Chekib Zedini, Rania Bannour, Imen Bannour, Badra Bannour, Majdi Jlassi, Leila Goul, Hedi Khairi

**Affiliations:** 1 Department of Family and Community Medicine, Faculty of Medicine, Sousse, 4000 Sousse, Tunisia,; 2 Faculty of Medicine Ibn Al Jazzar, University of Sousse, Sousse, Tunisia,; 3 Research Laboratory “LR12ES03”, 4002 Sousse, Tunisia,; 4 Department of Gynecology and Obstetrics, University Hospital Farhat Hached, Street Doctor Moreau, 4000 Sousse, Tunisia

**Keywords:** Grossesse gémellaire, accouchement, césarienne, score Apgar, Twin pregnancies, delivery, caesarean section, Apgar score

## Abstract

En dépit des progrès obstétricaux et pédiatriques, les grossesses gémellaires représentent une situation à haut risque aussi bien pour le déroulement de la grossesse que pour l´accouchement et reste encore une situation angoissante pour l´équipe obstétricale. Le but de cette étude était de décrire la pratique des accouchements des grossesses gémellaires au service de gynécologie obstétrique de Sousse, de décrire le pronostic maternel et fœtal et d´analyser les facteurs pouvant l´influencer. Nous avons réalisé une étude descriptive, rétrospective portant sur les accouchements des grossesses gémellaires sur une période de deux ans. Ont été incluses dans l´étude les grossesses gémellaires ayant atteint au moins 28 semaines d´aménorrhée (SA) et les femmes ayants une grossesse gémellaire compliquée d'une mort fœtale in utéro. Ont été exclues les femmes ayant une grossesse gémellaire et ayant accouchés avant 28 SA. Les grossesses bi-choriales bi-amniotiques représentaient 67% des cas, contre seulement 11,5% de grossesses mono-choriales bi-amniotiques et 3% de grossesses mono-choriale mono-amniotique. Nous avons recensé 52 césariennes programmées. Le travail a été spontané dans 304 cas. Il a été déclenché dans 43 cas. Au total 178 parturientes ont accouché par voie basse (44,6%), contre 215 par voie haute (53,9%). Le taux de césarienne pour le deuxième jumeau était de 1,5%. Quand l'accouchement était par voie basse, 19 cas de complications ont été observés (10,7%). Nous avons analysé le score d'Apgar du premier jumeau et du deuxième jumeau en fonction du mode d'accouchement. Il n'y a pas eu de différence statistiquement significative du score d'Apgar entre les deux voies d'accouchement. La morbi-mortalité périnatale est plus importante pour le deuxième jumeau que pour le premier jumeau. La morbidité maternelle en cas d´accouchement par voie basse était supérieur à la morbidité en cas d´accouchement par césarienne. Il n´y a pas de différences significatives dans le score d´Apgar selon que les enfants sont nés par voie basse ou par césarienne.

## Abstract (en)

Despite obstetric and pediatric progress, twin pregnancy still represents nowadays a high-risk situation for both pregnancy and childbirth. It still remains a distressing situation for the obstetric team. The aim of this study was to describe the practice of twin pregnancy deliveries in the obstetrics and gynecology department of Sousse, the maternal and fetal prognosis and to analyze the factors that may influence it. A descriptive, retrospective study of twin pregnancies births was conducted over a period of two years. We included twin pregnancies that have reached at least 28 weeks of amenorrhea (SA) and women having a twin pregnancy complicated by fetal death in utero. Women with a twin pregnancy who gave birth before 28 weeks were excluded. Bi-chorionic bi-amniotic pregnancies represented 67% of cases, compared with only 11.5% of mono-chorial bi-amniotic pregnancies and 3% of mono-chorial mono-amniotic pregnancies. A total of 52 patients have scheduled caesareans. Spontaneous labor was noticed in 304 cases, and was initiated in 43 cases. A total of 178 women gave birth by vaginal delivery (44.6%) and 215 by cesarean (53.9%). The cesarean rate for the second twin was 1.5%. When the delivery was vaginal, 19 cases of complications were observed (10.7%). We analyzed the Apgar score of both the first and the second twin according to the mode of delivery. There was no statistically significant difference in the Apgar score between the two delivery routes. Perinatal morbidity and mortality is higher for the second twin than for the first twin. Maternal morbidity was higher in vaginal delivery than cesarean delivery. There are no significant differences in the Apgar score according to whether the infants were born by vaginal delivery or by caesarean.

## Introduction

La fréquence des grossesses gémellaires a augmenté de manière importante ces dernières années [1] consécutive au recours de plus en plus fréquent aux techniques de procréation médicalement assistée [2-4]. En dépit des progrès obstétricaux et pédiatriques, les grossesses gémellaires représentent une situation à haut risque aussi bien pour le déroulement de la grossesse que pour l´accouchement et reste encore une situation angoissante pour l´équipe obstétricale, d´où la nécessité d´une surveillance médicale particulière dans des structures spécialisées [5,6]. L´accouchement gémellaire quel que soit son mode impose la présence d´une équipe multidisciplinaire et d´un bloc opératoire à proximité.

Cependant, le suivi de la grossesse gémellaire est actuellement assez mal codifié, et il existe une grande hétérogénéité de prise en charge périnatale et à l´accouchement. Le principal obstacle rencontré est la pauvreté d´informations scientifiques issues d´études de haut niveau de preuve permettant d´émettre des recommandations concernant l´accouchement des grossesses gémellaires [5]. C´est dans ce cadre que nous avons fait ce travail dans le but de rapporter l´expérience du service de gynécologie et obstétrique du centre hospitalo-universitaire Farhat Hached, en matière de l´accouchement des grossesses gémellaire durant une période de deux ans. Pour ce fait, nous nous sommes assigné les objectifs suivants: 1) décrire la pratique des accouchements des grossesses gémellaires au service de gynécologie obstétrique du CHU Farhat Hached de Sousse; 2) décrire le pronostic maternel et fœtal; 3) analyser les facteurs pouvant l´influencer.

## Méthodes

Nous avons réalisé une étude descriptive, rétrospective portant sur les accouchements de type grossesses gémellaires recensés à la maternité de l´hôpital universitaire Farhat Hached de Sousse, et ce sur une période de deux ans allant du 1^er^ janvier 2014 au 31 décembre 2015.

**Les critères d´inclusion:** ont été incluses dans l´étude: les grossesses gémellaires ayant atteint au moins 28 SA peu importe leurs origines ou le lieu de suivi de leurs grossesses. Les femmes ayants une grossesse gémellaire compliquée d'une mort fœtale in utéro.

**Les critères de non inclusion:** les femmes ayant une grossesse gémellaire et accouchés avant 28 SA. Les femmes ayant une grossesse triple au début réduite ensuite après la mort d´un fœtus. La collecte des données a été réalisée en utilisant une fiche de recueil. Nous avons recueilli les caractéristiques sociodémographiques des parturientes, les antécédents médicaux, chirurgicaux et obstétricaux maternels, les caractéristiques de la grossesse gémellaire les complications maternelles et néonatales au cours de l´accouchement. Une fois recueillies, les données ont été saisies et analysées à l´aide du logiciel Epi-Info 6. Les variables qualitatives, ont été représentées sous forme de fréquences absolues et relatives (pourcentages). Nous avons utilisé le test du Chi-deux pour la comparaison des variables qualitatives. Un seuil de signification de 5% a été retenu.

## Résultats

**Fréquence:** au cours de la période d'étude, 22552 accouchements ont été enregistrés au service de gynécologie obstétrique de l´hôpital Farhat Hached de Sousse dont 399 accouchements gémellaires. Ainsi le taux de grossesses gémellaires est de 1,76%.

**Caractéristiques des parturientes:** l'âge moyen des parturientes au moment de l'accouchement était de 30,7±5,2 ans avec des extrêmes allant de 19 à 45 ans. Les antécédents médicaux chirurgicaux et gynéco-obstétricaux de nos parturientes sont résumés dans le Tableau 1. Le terme “autres”, englobe 2 cas d'anémie, 2 cas d'asthme, 2 cas d'hépatite virale, un cas de lupus, un cas d'insuffisance rénale, un cas d'allergie à la pénicilline, un cas de pyélonéphrite et un cas de cardiopathie (insuffisance mitrale).

**Tableau 1:** caractéristiques des parturientes

Caractéristiques des parturientes		Nombre	Fréquence (%)
Antecedents médicaux	Hypertension artérielle	8	2
	Diabéte	2	0,5
	Hypothyroidie	6	1,5
	Autres	11	2,8
Gestité		1	38,8%
		2	22,3%
		3	18,3%
		4	10,8%
		≥5	9,8%
Parité		0	45;8%
		1	24,6%
		2	17%
		3	9%
		4	2,3%
		≥5	1,3%
Mode de conception	Grossesse spontannée	350	87,7%
	Grossesse induite	49	12,3%

**Le type de grossesse gémellaire (chorionicité/amniocité):** les grossesses bi-choriales bi-amniotiques représentaient 66,9% des cas dans notre population, contre seulement 11,5% de grossesses mono-choriales bi- amniotiques. Douze cas de grossesse mono-choriale mono-amniotique ont été recensés dans notre série (3%). Le type de grossesse gémellaire était non précisé dans 74 cas (18,5%).

**Le mode de déclenchement du travail:** le travail a été spontané dans 304 cas, soit 76,2%. Toutefois, le travail a été déclenché dans 43 cas. Les méthodes utilisées sont: un décollement du pole inférieur de l´œuf (14 cas), une amniotomie (8 cas), de l´ocytocine (21 cas). Par ailleurs, nous avons répertorié 52 césariennes faites avant le travail.

**Figure 1:**
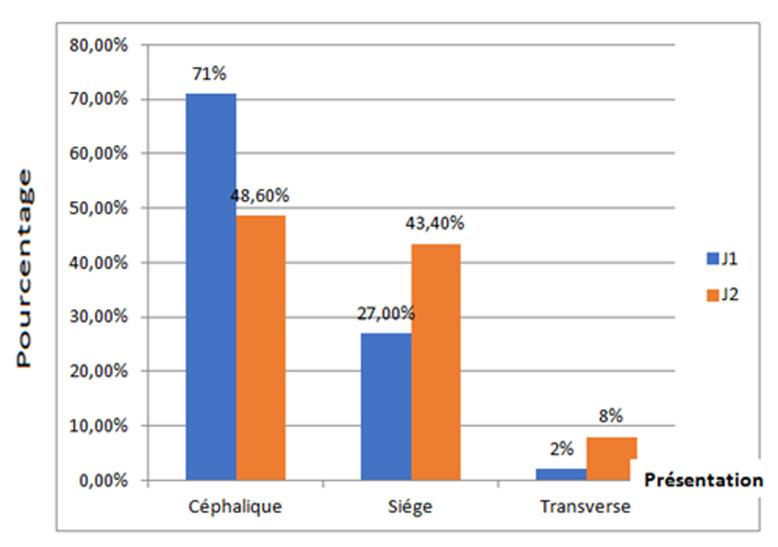
répartition des jumeaux selon leur ordre et leur présentation

**La présentation des jumeaux:** la présentation du premier jumeau était céphalique dans 71% des cas et la présentation du second jumeau était siège dans 43,4% des cas (Figure 1). La présentation des jumeaux n'a pas pu être précisée chez deux femmes. Il s'agit de la présentation de J1 dans le cas d'accouchements à domicile.

**Figure 2:**
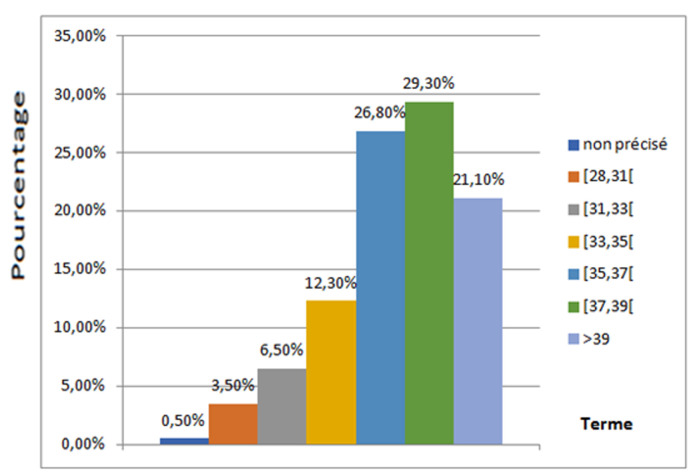
répartition selon le terme d'accouchement

**Le terme de l'accouchement:** le terme médian de l'accouchement était de 37 SA, avec des extrêmes allant de 28 SA à 42 SA+2 jours. Le terme d'accouchement était non précisé pour deux parturientes, l'une est une mère célibataire et l'autre, une grossesse non suivie (Figure 2).

**Figure 3:**
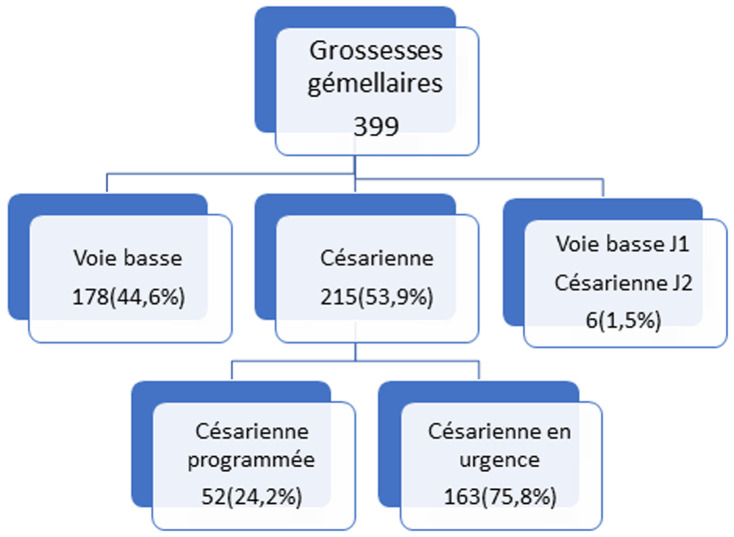
voies d´accouchement des grossesses gémellaires

**Les voies et les modalités d´accouchement:** cent soixante-dix-huit parturientes ont accouché par voie basse (44,6%), contre 215 par voie haute (53,9%) dont 52 césariennes programmées (13%). Le taux de césarienne pour le deuxième jumeau était de 1,5% (Figure 3) L'accouchement du premier jumeau a été fait par césarienne dans un peu plus d'un cas sur deux (53,9%) et celui par voie basse normale dans 44,4%. L'accouchement du deuxième jumeau a été fait par voie basse normal dans 43,1%. Une manœuvre a été faite sur le deuxième jumeau à chaque fois que sa présentation n'était pas céphalique.

### Le pronostic materno fœtal

**Pronostic maternel:** quand l'accouchement était par voie basse, 19 cas de complications ont été observés, soit un taux de 10,7%. On a noté 3 déchirures vulvo-périnéale (1,6%) et 16 cas d´hémorragie de la délivrance (9%). Pour les patientes qui ont accouché par césarienne, nous n'avons enregistré aucune complication.

**Pronostic fœtal: score d´Apgar:** dans notre étude, nous avons enregistré 778 naissances vivantes (391 pour J1 et 387 pour J2) et 20 morts fœtales in utéro. Le score d'Apgar a été évalué à la 1^è^^re^, 5^e^ et 10^e^ minute, un score inférieur à 7 a été considéré comme synonyme de souffrance fœtale aiguë (SFA) (Tableau 2). A la 1^ère^ minute, le score d´Apgar était significativement plus bas pour le deuxième jumeau. Trente-huit nouveau-nés ont eu un score d'Apgar à une minute inférieur à 7. Parmi ces derniers, 11 nouveau-nés ont gardé ce score à 5 minutes et 9 nouveau-nés l'ont gardé à 10 minutes. Un cas de décès de nouveau-né a été noté malgré la réanimation néonatale. Nous avons analysé le score d'Apgar du premier jumeau et du deuxième jumeau en fonction du mode d'accouchement. Il n'y a pas eu de différence statistiquement significative du score d'Apgar quel que soit la voie d'accouchement des deux jumeaux (Tableau 3).

**Tableau 2:** score d'Apgar des deux jumeaux à la 1^ère^, 5^e^ et 10^e^ minute

Score d'Apgar des deux jumeaux		J2 à la 1^ère^ minute		J2 à la 5^e^ minute		J2 à la 10^e^ minute		p
		Apgar<7	Apgar≥7	Apgar<7	Apgar≥7	Apgar<7	Apgar≥7	
J1 à la 1^ère^ minute	Apgar<7	10	8					<10^-3^ test exact de Fisher
	Apgar≥7	15	354					
J1 à la 5^e^ minute	Apgar<7			2	7			0,01 Test exact de Fisher
	Apgar≥7			5	373			
J1 à la 10^e^ minute	Apgar<7					2	7	0,005 Test exact de Fisher
	Apgar≥7					3	375	

**Tableau 3:** score d'Apgar en fonction du mode d'accouchement du premier et du deuxième jumeau

	Apgar	Mode d'accouchement J1		Mode d'accouchement J2		p
		Accouchement voie basse	Césarienne	Accouchement voie basse	Césarienne	
A la 1^è^^re^ minute	<7	2	11			0,02
	≥7	179	199			
A 5 minutes	<7	1	3			0,6
	≥7	180	207			
A 10 minutes	<7	1	3			0,6
	≥7	180	207			
A la 1^è^^re^ minute	<7			10	15	0,6
	≥7			164	198	
A 5 minutes	<7			2	5	0,6
	≥7			172	208	
A 10 minutes	<7			2	3	1
	≥7			172	210	

## Discussion

**Fréquence des grossesses gémellaires:** au cours de la période de notre étude, le taux de grossesses gémellaires était de 1,76%, un taux concordant avec ceux rapportés dans la littérature internationale, qui varient de 1,56% à 3,3% [7,8,9].

### Le travail et l'accouchement

**Déclenchement artificiel du travail:** le déclenchement du travail en cas de grossesses gémellaires peut s´avérer nécessaire afin de minimiser le risque de morbi-mortalité maternofœtale [10]. Nous avons eu recours à l´induction du travail dans 10,8% des cas, ce qui est en accord avec la littérature, où ce taux varie de 9,3% à 15,9% [10,11]. Les méthodes utilisées dans notre série étaient; un décollement du pole inférieur de l´œuf dans 32,6% des cas, une amniotomie dans 18,6% des cas et l´ocytocine dans 48,8% des cas. Dans la série récente de Ghassani [10], l´auteur comparait une cohorte de grossesses gémellaires bi-amniotiques (J1 céphalique), déclenchées ≥36 SA (n=156), à une cohorte de grossesses monofœtales déclenchées ≥36 SA (n=156). Le même protocole de déclenchement a été utilisé dans les 2 cohortes (ballonnet ± dinoprostone/ocytocine). Le taux de césarienne pour échec de déclenchement à terme (césarienne en phase de latence) était comparable entre les 2 populations (14,7% gémellaires contre 13,5% monofœtales; p=0,66). Ces constatations étaient également rapportées par d´autres auteurs pour les différentes modalités de déclenchement [11,12]. Ces auteurs concluaient que la gémellité n´apparaît pas comme facteur d´échec du déclenchement et que le protocole de déclenchement des grossesses monofœtales semble pouvoir être utilisé pour les jumeaux [10,11].

**Modalités de présentations de jumeaux:** au cours de l´accouchement, la présentation des jumeaux joue un rôle principal dans le choix de la voie d´accouchement, influençant de ce fait le pronostic fœtal et maternel [13]. La présentation céphalique-céphalique était la plus fréquente dans la littérature [14,15] comme dans notre série.

**Modalité d'accouchement:** le choix de la voie d´accouchement des grossesses gémellaires constitue toujours un sujet de débat depuis quatre décennies et il semblerait que la voie haute l´emporte. La nécessité du recours à une césarienne est augmentée en cas de grossesses gémellaires comparée aux singletons [16]. Dans notre série la césarienne était indiquée pour 53,9% des patientes, ce qui est concordant avec la plupart des auteurs. Cette voie d´accouchement est rapportée dans 18 à 72,3% dans la littérature [13,17,18].

### Etude du pronostic de l'accouchement des grossesses gemellaires

**Pronostic maternel:** l´accouchement d´une grossesse gémellaire représente une situation à risque accru de complication maternelle [5]. Ces complications ont survenu chez 6,5% patients dans notre série, une fréquence comparable à celle dans la série de Stach *et al*. [19] et d´Attah *et al*. [20], qui rapportent un taux de 6,9% et de 10% respectivement. Dans notre série l´hémorragie du post partum a été notée dans 4% des cas, ce qui est concordant avec les données de la littérature où sa fréquence est de 2,9% à 4,2% [19]. Les femmes ont un risque accru de mortalité en cas de grossesse gémellaire. Une étude européenne a montré que le taux de mortalité maternelle (pendant la grossesse, au moment de l´accouchement et dans les 24 jours suivants) était trois fois plus élevé en cas de grossesse multiple qu´en cas de grossesse unique [21].

**Pronostic fœtal:** l´évaluation de la voie d´accouchement la moins risquée pour les fœtus en cas de grossesse gémellaire est sujet d´un nombre important d´études. Dans une étude de cohorte sur les facteurs déterminant de la mortalité et la morbidité de J2, menée sur 1542 paires de jumeaux, Armson *et al*. [22] trouvait que les deuxièmes jumeaux à terme étaient plus à risque de morbidité après naissance par voie basse (RR 3.0, IC 95% 1.47-6.11) que par césarienne programmée (RR 1.0, IC 95% 0.14-7.10). Cependant, ces résultats n´étaient pas confirmés dans la méta-analyse plus récente sur ce sujet, menée par Rossi *et al*. [23], incluant 39 571 grossesses gémellaires dans 18 études, ou la morbidité néonatale était définie par un pH ombilical < 7.0, un score d´Apgar < 7 à 5 minutes et par le survenu d´un traumatisme néonatal. Pour le premier jumeaux, la morbidité néonatale était moins fréquente après naissance par voie basse (1,1%) par rapport à la naissance par césarienne (2.2%; OR 0.47; IC 95% 0.27-0.82). Pour le deuxième jumeaux, la césarienne sur J2 était plus pourvoyeuse de morbidité (19.8%) que la voie basse (9.5%; OR 0.55; IC 95% 0.41-0.74) ou la césarienne (9.8%; OR 0.47; IC 95% 0.43-0.53).

Si ces données n´ont que suggéré un effet délétère de la voie basse, ou de la césarienne, la nette surmorbidité de J2 en cas de dissociation de la voie d´accouchement n´est plus sujet de controverse. Dans notre série, il n'y avait pas de différence statistiquement significative du score d'Apgar quel que soit la voie d'accouchement des deux jumeaux. La surmorbidité du deuxième jumeau par rapport au premier jumeau a été bien documentée dans la littérature [24]. Le taux de morbidité est rapporté à 3% pour le premier jumeau contre 4,6% pour le deuxième dans la méta analyse de Rossi [23]. Dans la série d´Armson *et al*. [22] cette sur morbidité du deuxième jumeau était démontrée indépendamment de l´âge gestationnel, de la présentation; de la chorionicité ou du sexe des nouveau-nés (RR 1.62, IC 95% 1.38-1.9). Dans notre série, un score d´Apgar bas à la 1^ère^, 5^e^ et 10^e^ minute était plus fréquemment retrouvé chez le deuxième jumeau de manière significative.

## Conclusion

L´accouchement des grossesses gémellaires constituent un facteur de risque significatif de morbi-mortalité maternelle et périnatale par rapport aux grossesses simples. La morbi-mortalité périnatale est encore plus importante pour le deuxième jumeau que pour le premier jumeau. Dans notre étude, la morbidité maternelle en cas d´accouchement par voie basse était supérieure à la morbidité en cas d´accouchement par césarienne. Il n´y a pas de différences significatives dans le score d´Apgar selon que les enfants sont nés par voie basse ou par césarienne.

### Etat des connaissances sur le sujet

- Les grossesses gémellaires représentent une situation à haut risque aussi bien pour le déroulement de la grossesse que pour l´accouchement;
- La morbi-mortalité maternelle et périnatale est supérieure à celle de la grossesse unique.

### Contribution de notre étude à la connaissance

- La morbi-mortalité périnatale est encore plus importante pour le deuxième jumeau que pour le premier jumeau;
- Dans notre étude, la morbidité maternelle en cas d´accouchement par voie basse était supérieur à la morbidité en cas d´accouchement par césarienne;
- Il n´y a pas de différences significatives dans le score d´Apgar selon que les enfants sont nés par voie basse ou par césarienne.
